# Live birth, cumulative live birth and perinatal outcome following assisted reproductive treatments using donor sperm in single women vs. women in lesbian couples: a prospective controlled cohort study

**DOI:** 10.1007/s10815-022-02402-6

**Published:** 2022-02-01

**Authors:** Tove Wrande, Berglind Harper Kristjansdottir, Panagiotis Tsiartas, Nermin Hadziosmanovic, Kenny A. Rodriguez-Wallberg

**Affiliations:** 1 Department of Obstetrics and Gynecology, Nyköping Hospital, Nyköping, Sweden; 2 Department of Reproductive Medicine, Division of Gynecology and Reproduction, Karolinska University Hospital, 141 86 Stockholm, Sweden; 3 Department of Obstetrics and Gynecology, Sahlgrenska University Hospital, Gothenburg, Sweden; 4 Department of Obstetrics and Gynecology, Institute of Clinical Sciences, Sahlgrenska Academy, University of Gothenburg, Gothenburg, Sweden; 5 Uppsala Clinical Research Center, Uppsala University, Uppsala, Sweden; 6 Department of Oncology-Pathology, Karolinska Institute, Stockholm, Sweden

**Keywords:** Assisted reproductive technology, Sperm donor, Insemination, In vitro fertilization, Single women, Lesbian couples

## Abstract

**Purpose:**

Assisted reproductive technology (ART) treatments with donor sperm have been allowed for women in lesbian relationships (WLR) since 2005 in Sweden, but for single women (SW), these became approved only recently in 2016. This study was conducted to compare the outcomes of ART treatments in SW vs. WLR.

**Methods:**

This is a prospective controlled cohort study of 251 women undergoing intrauterine insemination (D-IUI) or in vitro fertilization (D-IVF) using donor sperm between 2017 and 2019 at the department of Reproductive Medicine, Karolinska University Hospital. The cohort comprised 139 SW and 112 WLR. The main outcomes included differences in live birth rate (LBR) and cumulative live birth rate (cLBR) between the groups. The SW underwent 66 D-IUI and 193 D-IVF treatments and WLR underwent 255 D-IUI and 69 D-IVF treatments. Data on clinical characteristics, treatment protocols and clinical outcomes were extracted from the clinic’s electronic database. The outcomes of D-IUI and D-IVF were separately assessed.

**Results:**

The cohort of SW was significantly older than WLR (37.6 vs. 32.4 years, *P* < 0.001), and more commonly underwent IVF at first treatment (83% vs. 29%, *P* < 0.000). Conversely, WLR underwent more frequently D-IUI as a first treatment (71% vs. 17% of SW, *P* < 0.001) and more often in the natural cycle (89.9% vs. 70.8%, *P* = 0.019), respectively. There was no statistically significant difference in the main outcome LBR between the two groups, or between the two different types of treatment, when adjusted for age. Perinatal outcomes and cLBR were also similar among the groups.

**Conclusions:**

SW were, on average, older than WLR undergoing treatment with donor sperm. No significant differences were seen in the LBR and cLBR when adjusted for age between the two groups and between the two types of treatment (D-IVF vs. D-IUI).

**Trial registration:**

ClinicalTrials.gov NTC04602962.

## Introduction

The use of donated sperm was introduced in reproductive medicine during the twentieth century and initially offered to heterosexual couples with male factor infertility, through intrauterine insemination (IUI) [1]. This technique became available even for single women (SW) thereafter and women in lesbian relationships (WLR). Moreover, using in vitro fertilization (IVF) and intracytoplasmic sperm injection (ICSI) techniques, the number of sperm cells required for treatment has dramatically decreased, and the efficiency of treatments using cryopreserved donor sperm is currently high [2]. The cause for the use of donor sperm in fertility treatments in SW and WLR is social, so one could assume treatment outcomes between the groups to be similar, but there are only a few studies performed in this matter. Nordqvist and co-workers found no difference in live birth rates (LBR) between heterosexual and WLR undergoing D-IUI and D-IVF [3]. However, in the studies of Fredriksen-Goldsen et al., Eliason et al. and Agrawal et al., it has been found a higher prevalence of conditions associated with reduced fertility, for example, polycystic ovary syndrome (PCOS), high BMI and high consumption of alcohol and tobacco among WLR [4–6]. A recently published meta-analysis has however yielded conflicting results, as it could not demonstrate any difference in the prevalence of endometriosis or PCOS between heterosexual and lesbian women [7]. Since single mothers by choice is a new patient group in the Swedish public health care system, there is still limited information on outcomes of assisted reproductive technology (ART) treatments in this group. The aim of this study was to prospectively assess whether there are differences in treatment outcomes and clinical and perinatal outcomes among SW and WLR undergoing D-IUI and D-IVF.

## Materials and methods

### Study design

In this single-centre, prospective controlled cohort study, we included all SW and WLR, who underwent D-IVF and D-IUI at the Department of Reproductive Medicine at the Karolinska University Hospital from 2017 to 2019, following the amendment in the Swedish law that allowed ART treatments to SW. SW and WLR that seek for treatment with sperm donation (SD) are required to fulfil strict medical and social criteria. The recipient of SD has to be between 25 and 40 years of age and have a body mass index (BMI) between18 and 35. Furthermore, SW or WLR must not have any social or psychological contraindications to parenthood. The recipient’s own oocytes were used for the SD treatments. During the study period, double donation was not legal in Sweden, and it was mandatory for the recipient of SD, and not her partner in the case of WLR, to receive all embryos. To be approved for treatment, all women must first undergo at least one medical examination and two appointments with a psychologist, for the required medical and psychosocial evaluation. The treatments are provided within the tax-financed healthcare available to the whole population with a limit of 6 D-IUIs or 3 D-IVFs to achieve a first child. Additional treatments required, or those attempting siblings are not covered by the tax-funded system, and the patients should pay for those. Donated sperm was retrieved from anonymous donators aged 23–45 years who underwent at least one medical and psychosocial evaluation, and the sperm quality was validated before used in SD treatments. The only data from the sperm donator that are known are eye, skin and hair colour, weight and height and were used for the matching with the SD recipient.

**Fig. 1:**
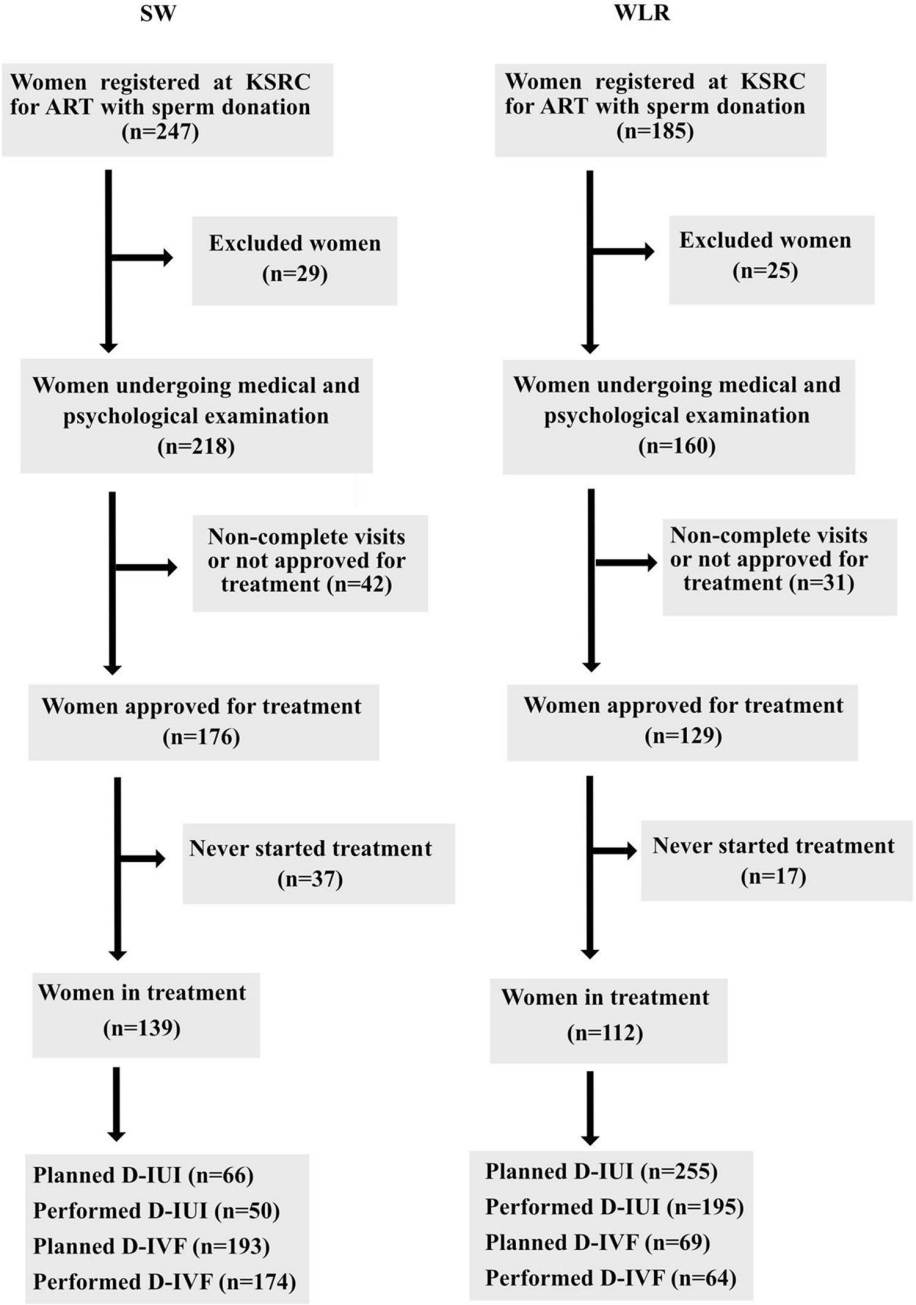
Flowchart over the inclusion of women in the study

For this study, clinical data including ART treatments and perinatal outcomes were retrieved from the reproductive centre’s electronic database. During the study period, 251 women underwent treatment with sperm donation as SW (*n* = 139) or as WLR (*n* = 112). The number of women going through each step towards fulfilling the treatment requirements is summarized in Fig. 1.

### Statistics

As two-thirds (*n* = 177/251) of the women included in the study had more than one treatment performed, we have analysed our data in a generalized estimating equation (GEE) model that addressed error estimates in the context of correlated observations, and therefore fully accounted for any dependence between treatments for the same individual [8]. The GEE model was used for dichotomous as well as for continuous outcomes. Results are reported as mean ± 95% CI for continuous variables and proportion (%) and 95% CI for categorical variables. *P* values from the GEE model are reported both unadjusted and age adjusted. Age was analysed as a continuous variable. A two-sided *P* value of less than 0.05 was considered significant. All statistical analyses were performed using SAS software version 9.4 (SAS Institute, Cary, NC).

## Results

**Fig. 2:**
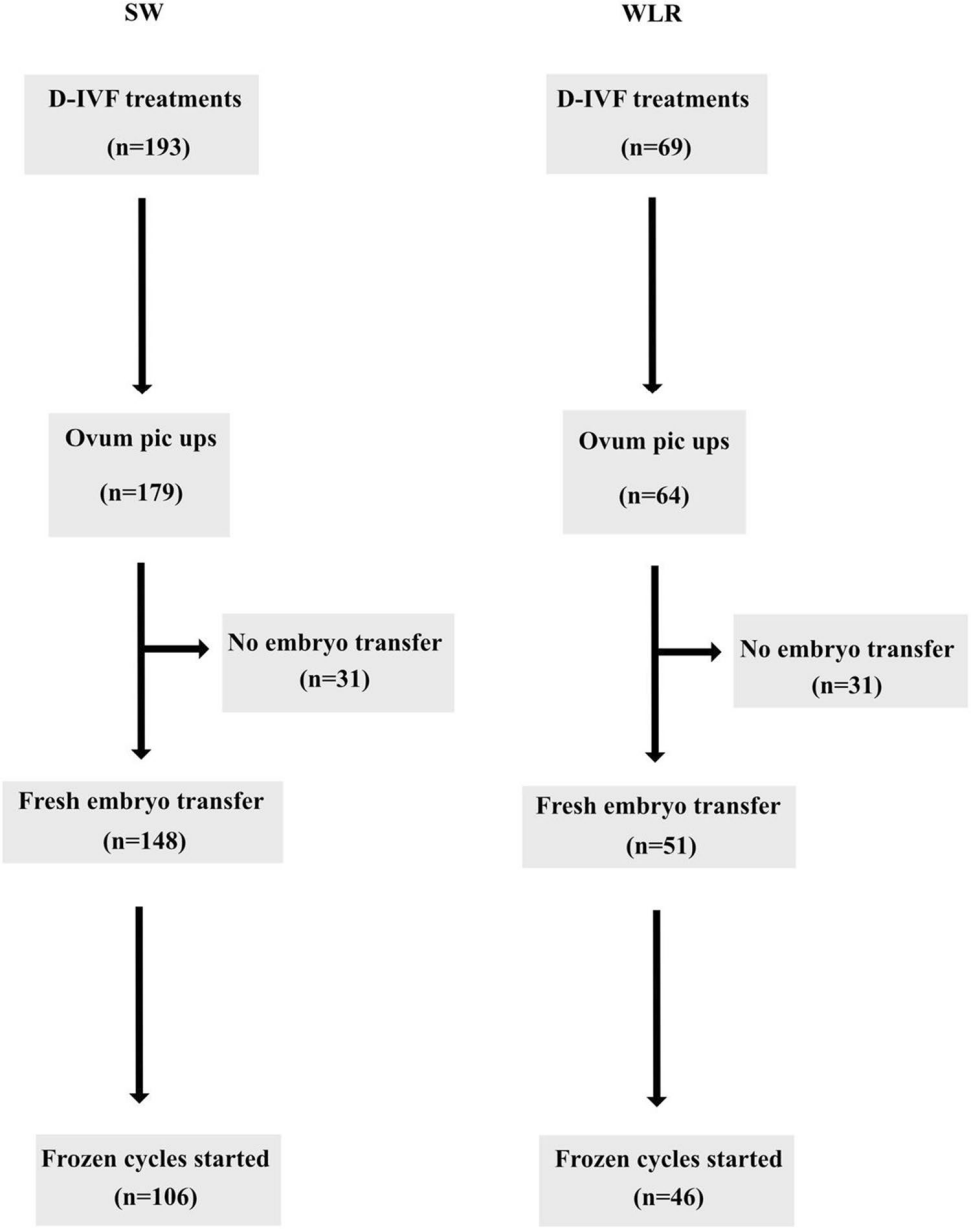
Flowchart over D-IVF treatments

In total, 321 D-IUI treatments were planned, and 245 performed among SW (*n* = 50) and WLR (*n* = 195) (Fig. 1). Insemination treatments were cancelled (*n* = 76) mainly due to anovulation (*n* = 28) or ovulation during the weekend (*n* = 27), as the centre does not perform IUI treatments during the weekends [9]. The number of D-IVF treatments performed among the two groups of women is shown in Fig. 2. SW were significantly older at the start of treatment (37.6 vs. 32.4 years, *P* < 0.001) and underwent more often conventional IVF at the first IVF treatment, when compared to WLR (82.7% vs. 28.6%, *P* < 0.001) (Table 1). In contrast, the WLR more often underwent D-IUI as the first treatment, compared with SW (71.4% vs. 17.3%, *P* < 0.001) (Table 1). WLR underwent more D-IUI cycles/woman compared to SW (3.1 vs. 2.3, *P* = 0.024). Natural cycle D-IUIs were more often performed among WLR compared with SW (89.9% vs. 70.8%, *P* = 0.019), whereas letrozole stimulated cycles were more often performed among the group of SW (18.5% vs. 8.4%, *P* = 0.043) (Table 2). In total, 36 children were born after 321 D-IUIs, *n* = 6 in SW and *n* = 30 in WLR. No differences were seen after D-IUI in treatment characteristics, cumulative live birth rate (cLBR) and perinatal outcomes between the two groups of women after adjusting for age (Table 2). In the group of WLR who underwent D-IVF compared with SW, a significantly higher cumulative clinical pregnancy rate per ovum pick-up (OPU) (58.1% vs. 34.3%, *P* = 0.002) and per embryo transfer (ET) (66.7% vs. 39.9%, *P* = 0.005) were seen after adjustment for age. In total, 35 children were born after D-IVF among both research groups, *n* = 21 in SW and *n* = 14 in WLR. In the group of WLR, a higher cumulative live birth rate per OPU (38.7% vs. 20.8%, *P* = 0.006) and per ET (45.1% vs. 24.3%, *P* = 0.006) was seen, but the results are not statistically significant after adjustment for age. No differences were seen in treatment characteristics, cLBR and perinatal outcomes between the groups after adjustment for age at treatment start (Table 3).

**Table 1:** Demographic and treatment characteristics based on index treatment. Results are presented as mean (95% CI) for continuous variables and proportion (%) (95% CI) (based on normal approximation method) for categorical variables. Statistically significant results are marked in bold

	SW (*n* = 139, 55.4%)	WLR (*n* = 112, 44.6%)	*P* value (crude)	*P* value (age adjusted)
Age at treatment start (years)	37.6 (37.2–37.9)	32.4 (31.9–33.0)	** < 0.000**	-
BMI	23.6 (22.9–24.3)	23.8 (22.9–24.7)	0.712	0.629
AMH (µg/L)	2.3 (1.9–2.7)	3.2 (2.7–3.6)	**0.004**	0.812
TSH (mIU/L)	1.7 (1.5–1.9)	1.9 (1.7–2.0)	0.131	0.066
AFC	13.7 (12–15.5)	19.3 (17.3–21.3)	** < 0.000**	0.688
Smoker	2.7 (0–6.4)	2.5 (0–5.9)	0.937	0.849
Snuffer	12.0 (4.5–19.5)	9.9 (3.2–16.5)	0.671	0.568
Conventional IVF	82.7 (76.4–89.1)	28.6 (20.1–37.1)	** < 0.000**	**0.000**
ICSI	13.5 (6.8–20.1)	7.4 (0–18.0)	0.399	0.204
D-IUI	17.3 (10.9–23.6)	71.4 (62.9–79.9)	** < 0.000**	**0.000**
Natural cycles (D-IUI)	41.7 (20.4–62.9)	56.3 (45.1–67.4)	0.212	0.255

*SW* single women, *WLR* women in lesbian relationship, *AMH* anti-Müllerian hormone, *TSH* thyroid stimulating hormone, *AFC* antral follicle count, *IVF* in vitro fertilization, *ICSI* intracytoplasmic sperm injection

**Table 2:** Treatment outcomes for D-IUI. Results are presented as mean (95% CI) for continuous variables and proportion (%) (95% CI) for categorical variables. *P* values calculated from generalized estimating equation (GEE) model. Cumulative outcomes were calculated through taking into account all treatments (fresh and frozen) that every woman has gone through during the data collection period. Statistical significant results are marked in bold

	Treatments in SW (*n* = 66, 20.6%)	Treatments in WLR (*n* = 255, 79.4%)	*P* value (crude)	*P* value (age adjusted)
Natural cycles	70.8 (59.4–82.1)	89.9 (85.9–93.8)	**0.029**	**0.019**
Letrozole stimulated cycles	18.5 (8.8–28.2)	8.4 (4.7–12.0)	0.222	**0.043**
FSH/hMG stimulated cycles	9.2 (2.0–16.5)	0.4 (0–1.3)	**0.004**	0.589
FSH/hMG stimulation (days)	11.3 (9.6–12.9)	14.2 (12–16.4)	**0.038**	0.111
Total FSH/hMG dose (IU)	1111 (221.5–2000)	423.3 (0–1069)	0.249	0.218
Cancelled cycles	24.2 (13.6–34.9)	23.5 (18.3–28.8)	0.903	0.577
D-IUI cycles/woman	2.3 (1.7–2.9)	3.1 (2.7–3.5)	**0.011**	**0.024**
CPR/started cycle	15.2 (6.3–24.0)	19.2 (14.3–24.1)	0.420	0.969
CPR/insemination	20 (8.5–31.5)	25.1 (19–31.3)	0.419	0.897
Cumulative CPR/person	35.7 (16.8–54.6)	53.8 (42.6–64.9)	0.103	0.879
Miscarriage rate	30 (0–64.6)	26.5 (13.7–39.3)	0.828	0.782
LBR/started cycle	10.6 (3–18.2)	14.1 (9.8–18.4)	0.470	0.744
LBR/insemination	14 (4–24)	18.5 (13–24)	0.481	0.699
Cumulative LBR/person	25 (7.9–42.1)	43.8 (32.6–54.9)	0.084	0.827
Gestational age at delivery (weeks)	36.5 (32.3–40.8)	37.5 (36.7–38.3)	0.474	0.507
Birth weight (g)	3218 (2568–3868)	3351 (3067–3634)	0.588	0.896
Birth length (cm)	50 (47.2–52.8)	49.4 (47.8–51)	0.592	0.590
Vaginal delivery	66.7 (12.5–120.9)	60 (41.4–78.6)	0.761	0.974
Caesarean section	16.7 (0–59.5)	33.3 (15.4–51.2)	0.431	0.490

*SW* single women, *WLR* women in lesbian relationship, *hMG* human menopausal gonadotropin, *FSH* follicle stimulating hormone, *CPR* clinical pregnancy rate, *LBR* live birth rate

**Table 3:** Treatment outcomes for D-IVF. Results are presented as mean (95% CI) for continuous variables and proportion (%) (95% CI) for categorical variables. *P* values calculated from generalized estimating equation (GEE) model. Cumulative outcomes were calculated through taking into account all treatments (fresh and frozen) that every woman has gone through during the data collection period. Statistical significant results are marked in bold

	Treatments in SW (*n* = 193, 73.7%)	Treatments in WLR (*n* = 69, 26.3%)	*P* value (crude)	*P* value (age adjusted)
FSH/hMG stimulation (days)	10.5 (10.1–10.8)	10.1 (9.7–10.5)	0.234	0.862
Total FSH/hMG dose/OPU	2911 (2700–3122)	2271 (1996–2546)	**0.001**	0.637
No. of oocytes/OPU	7.5 (6.7–8.3)	9.7 (8–11.4)	**0.023**	0.302
No. of obtained embryos	4.5 (3.9–5)	6.3 (5.1–7.6)	**0.009**	0.132
No. of frozen embryos	1.4 (1.1–1.7)	2.7 (2–3.4)	**0.001**	0.705
SET	99.3 (97.9–100)	100 (-)	-	-
No. of vitrified embryos	1.1 (0.8–1.4)	2.1 (1.4–2.9)	**0.009**	0.75
Cancelled cycles before OPU	23.3 (17.3–29.3)	26.1 (15.5–36.7)	0.655	0.251
D-IVF cycles/woman	1.6 (1.5–1.8)	1.5 (1.2–1.7)	0.2	0.76
Frozen D-IVF cycles/woman	1.39 (1.09–1.7)	2.69 (1.97–3.41)	**0.001**	0.7
No. of remaining frozen embryos/woman	1.06 (0.78–1.36)	2.14 (1.42–2.86)	**0.008**	0.75
CPR/stimulation start	22.8 (16.8–28.8)	36.2 (24.6–47.9)	**0.035**	0.102
CPR/OPU	24.7 (18.3–31.1)	40.3 (27.8–52.9)	**0.024**	0.105
CPR/fresh ET	29.7 (22.3–37.2)	49 (34.8–63.2)	**0.017**	0.203
CPR/FET	38.3 (25.7–51)	38.9 (22.2–55.6)	0.957	0.614
Cumulative CPR/OPU	34.3 (27.2–41.3)	58.1 (45.4–70.7)	**0.001**	**0.002**
Cumulative CPR/ET	39.9 (31.9–47.8)	66.7 (53.3–80.1)	**0.000**	**0.005**
Miscarriage rate	52.3 (36.9–67.6)	36 (15.8–56.2)	0.195	0.714
LBR/stimulation start	10.9 (6.4–15.3)	23.2 (13–33.4)	**0.018**	0.144
LBR/OPU	11.8 (7–16.6)	25.8 (14.6–37)	**0.013**	0.159
LBR/fresh ET	14.2 (8.5–19.9)	31.4 (18.2–44.6)	**0.011**	0.259
LBR/FET	26.7 (15.1–38.2)	22.2 (8–36.5)	0.642	0.425
Cumulative LBR/OPU	20.8 (14.8–26.8)	38.7 (26.2–51.2)	**0.006**	0.054
Cumulative LBR/ET	24.3 (17.3–31.3)	45.1 (31–59.2)	**0.006**	0.088
Gestational age at delivery (weeks)	37.7 (37–38.3)	36.4 (33–39.8)	0.380	0.378
Birth weight (g)	3595 (3368–3821)	3576 (2976–4176)	0.932	0.615
Birth length (cm)	50.8 (49.8–51.8)	50.2 (48.6–51.8)	0.390	0.641
Vaginal delivery	50 (24.4–75.6)	50 (20–80)	1.000	0.894
Instrumental delivery	11.1 (0–27.2)	7.1 (0–22.6)	0.704	0.762
Caesarean section	22.2 (0.9–43.5)	28.6 (1.5–55.6)	0.681	0.267

*SW* single women, *WLR* women in lesbian relationship, *FSH* follicle stimulating hormone, *OPU* ovum pick-up, *SET* single embryo transfer, *CPR* clinical pregnancy rate, *LBR* live birth rate

## Discussion

In this study, we compared the clinical and perinatal outcomes between SW and WLR that underwent D-IUI and D-IVF treatments at a single centre between 2017 and 2019. We did not find any difference for the primary outcome LBR, and there were no differences in cumulative LBR or perinatal outcomes when comparing the two groups according to treatment.

Arguments against the rights of SW and lesbian couples to become parents have mainly been based on concerns for the wellbeing of the future child that would grow up without a father figure, and this has been regarded as detrimental to the child’s psychosocial development. A previous study that assessed the health of children and adolescents growing with a single mother due to divorce, separation or step parenting showed negative outcomes (adjustment problems and lower prosocial scores) in these children. However, the study did not investigated mother-headed families by SW or lesbian couples [10]. Conditions like ongoing conflicts and economic hardship are assumed to affect children to a larger extent when it comes to developing adverse behaviours, than growing up with a single parent [11]. The psychological development of children of single mothers by choice showed that the quality of family relationship is important for the well-being of the children [12, 13]. Moreover, a study showed that children growing up in lesbian couples function well when entering adulthood [14]*.* The current knowledge on single, lesbian and heterosexual women undergoing fertility treatment with donated sperm cells is however still contradictory and limited. According to Hudson et al., the ART success rate was higher in lesbian women in comparison to heterosexual women [15]. However, other studies have not found any differences in clinical outcome between single mothers, lesbian and heterosexual women. Ferrara et al. studied SW vs. lesbian women undergoing D-IUI and showed no difference in pregnancy rate after age adjustment [16]. A more recent study that compared SW, lesbian and heterosexual couples found no difference in pregnancy rates after D-IUI treatments [17]. In studies showing higher pregnancy rates in lesbian couples compared to heterosexual couples, it is sometimes difficult to see if confounders for infertility in heterosexual couples undergoing ART compared to lesbian couples with social infertility are considered which make it difficult to interpret study results [18]. In Sweden, the use of donated sperm for IUI in married couples was regulated by law in 1985 [19] and for IVF in 2003 [20]. Lesbian couples have been offered the same access to ART treatments since 2005 [21]. An amendment in legislation was approved 2016, offering ART treatments even to SW within the public healthcare system [22]. At Karolinska University Hospital, the first single woman wishing to undergo ART treatment as a single mother by choice started her medical and psychosocial evaluation in November 2017, and the first ART treatment with donated sperm was performed in January 2018.

Increasing female age is one of the most important factors for the declining fertility in couples [23, 24]. Single mothers by choice tend to seek ART treatments later in life, when they are more socially established, with negative consequences for their fertility outcomes due to age-related infertility. The group of women in lesbian couples in this study was generally younger and had better ovarian reserve (based on anti-Müllerian hormone (AMH) levels and antral follicle count (AFC)) when compared to SW, which was a determining factor in the choice of D-IUI, as opposed to D-IVF, as the first attempted ART treatment in the group of SW. Other important factors influencing fertility (BMI, nicotine habits) were comparable between the two groups. The group of SW underwent D-IUI in a letrozole stimulated cycle significantly more often compared to women in lesbian couples. This could be due to the older age and the reduced ovarian reserve among the group of SW, thus requiring treatment to medically induce ovulation. Letrozole was the main medicine for ovulation induction that was used at the reproductive centre at Karolinska University Hospital due to its effectiveness and safety [25]. Alternative methods for ovulation induction before D-IUI were used more often among SW compared with the lesbian women in this study were follicle stimulating hormone (FSH), and human menopausal gonadotropin (hMG) were used instead of letrozole. All these cases in this study were planned D-IVF treatments converted to D-IUI because of poor response to stimulation and not a deliberate different treatment protocol for SW. Almost one-fourth of all D-IUI treatments were cancelled in both groups, mainly due to collision with weekends when no IUIs were performed at the clinic or due to the lack of luteinizing hormone (LH) peak detected by the ovulation test. However, the cumulative perinatal outcomes after D-IUI did not differ significantly between the groups.

In this study, SW were more likely to undergo D-IVF as the first ART treatment rather than D-IUI, usually because of the higher age at the start of treatment. Women, older than 38 years, are often recommended to attempt IVF directly, due to the higher expected efficacy of this treatment when compared to IUI. Although many pregnancies were still ongoing when data were analysed in the D-IVF group, the rate of ongoing clinical pregnancy was significantly higher in the group of women in lesbian couples compared with the group of SW. The lower cumulative success rates of D-IVF among SW were reflected by the higher total dose of FSH per cycle needed for ovarian stimulation, lower number of retrieved oocytes and embryos and lower number of frozen embryos. Nevertheless, SW were older and may have undergone fewer treatments compared to the younger group of women in a lesbian couple that more often have longer time available for more attempts. Previous studies have shown a slightly higher risk for adverse perinatal and obstetrical outcomes after IVF compared to natural conceptions [26, 27]. The cumulative LBR after D-IVF for SW was significantly lower compared to lesbian women. However, after adjusting for the age difference between the groups, this difference in cumulative LBR disappeared. However, the small size of the study population and the fact that some of the pregnancies were still ongoing during data collection and analysis must be taken in consideration as possible biasing factors for the outcome.

The strengths of our study are the large amount of included health parameters and socioeconomical factors that are known to affect the outcome of ART such as age, smoking habits, BMI, AMH and AFC. Additionally, the results were age-adjusted in order to exclude the negative impact of increased age on the outcome of the performed treatments. Limitations include the small number of women included in the study. However, the highest possible number of women was included during the study period since Karolinska University Hospital was at the time of the study inclusion the only reproductive centre in Stockholm offering D-IVF to SW. In order to overcome this problem, a multicentre study or a longer time interval to include more patients would be preferable for future studies. Another possible limitation is that many women were still pregnant or had an ongoing ART treatment when the study period ended which limited the access to the treatment outcome data for all women. Additionally, many women underwent both D-IUI and D-IVF and this could render it difficult to calculate the cumulative treatment outcomes. Another possible limitation is that in the group of women in lesbian couples, approximately 40 of the treatments were performed on women with previous children for sibling treatments. Previous childbearing is known to be favourable for future fertility, and a sub-analysis of the data, where women with previous children would be excluded, could overcome this limitation. This was not a limitation for the group of SW as nulliparity was a requirement to be accepted for ART as single. A further limitation is that some of the patients may have previously undergone D-IUIs in other clinics, and those treatments could not be included in this study.

## Conclusion

The likelihood of having a child with ART treatments using donated sperm was similar between the group of SWs when compared to the group of WLR, when adjusted for age. Perinatal outcomes and cLBR were also similar among the groups.

Although the SW were generally older during the treatment, when compared to WLR, they still had a good chance of achieving pregnancy and childbirth after treatment with donated sperm. These results support the opinion that SW should be offered ART within the public healthcare system as they benefit as much as other women who require donated sperm to conceive. These non-standard situations are relatively new in reproductive medicine, and the evidence on the success rate of ART treatments for these groups is still limited. Since ART treatments are costly and sometimes psychologically challenging, it would be beneficial to have more information on the success of these treatments so that patients can be correctly advised.

## Data Availability

The datasets generated and/or analysed during the current study are available from the corresponding author on reasonable request.
